# Family-centered care

**DOI:** 10.1186/1824-7288-40-S1-A33

**Published:** 2014-08-11

**Authors:** Filippo Festini

**Affiliations:** 1 Department of Health Sciences, University of Florence, Nursing Research Unit, Meyer Children Hospital, Florence, 50139, Italy

## 

Family-Centered Care (FCC) is a way to provide health care that recognizes the importance of family for a hospitalized child. According to it, it is essential to involve families in their children’s recovery plan [1].

FCC was born in the Fifties of 20th century as a reaction to the leading idea in Pediatric care that emphasized physical care, technical abilities and emotional detachment and considered the family as a potential threat to a child’s path to recovery. Thus, families -particularly mothers- were physically separated from their sick children, being “substituted” by healthcare professionals, who took on parents’ caring functions.

FCC is aimed to maintain and strengthen family roles and the natural bond the family has with the child, in order to promote a healthy family functioning, even through sickness. The physical presence of the family at bedside was FCC’s first achievement [2].

Some Authors define FCC as: “the professional support to the child and its family, through participation, engagement and sharing, in a contest of empowerment and negotiation” [1].

According to some Authors, there are eight core concepts that accomplish FCC in everyday clinical practice: 1- to consider family to be the centerpiece of a child’s life, remembering that healthcare professionals and healthcare delivery structures should be temporary entities, thus they have to "enter on tiptoe" in a little patient’s own world; 2- to promote cooperation between families and healthcare professionals; 3- to facilitate communication and a bidirectional exchange of information between families and healthcare professionals, with no omissions, nor falsifications; 4- to recognize and respect each family’s peculiarities (cultural, ethnic, spiritual etc) and its strengths; 5- to recognize and respect each family’s coping strategies, to value them and to include these strategies in every recovery plan; 6- to encourage support between families; 7- to assure the best and highest grade of flexibility and accessibility when providing health care services, particularly for those families whose children need specialized and chronic therapies;8- To always care about emotions, fears and desires of the family [3].

Even though FCC is the common gold standard philosophy of health care in the pediatric departments of Western industrialized countries, it has recently been introduced in Italy, where FCC is still uncommon.

In order to evaluate to what extent FCC is implemented in a hospital, a FCC questionnaire is available [4].
